# Treatment of a Gastric Lactobezoar with N-Acetylcysteine

**DOI:** 10.1155/2014/254741

**Published:** 2014-11-23

**Authors:** Brandon Sparks, Anil Kesavan

**Affiliations:** ^1^Department of Pediatrics, Rush University Medical Center, Jones Building, 1653 W Congress Pkwy, Suite 770, Chicago, IL 60612, USA; ^2^Section of Pediatric Gastroenterology, Rush University Medical Center, Professional Building, 1725 W Harrison Street, Suite 710, Chicago, IL 60612, USA

## Abstract

Lactobezoars are a rare finding with potentially serious sequelae in pediatric patients with feeding intolerance. Aggressive treatment may be preferred to traditional treatments to avoid complications in medically complex patients. In our patient, N-acetylcysteine lavage was a safe and effective alternative that resulted in rapid resolution of his feeding intolerance.

## 1. Introduction

Feeding intolerance is a common finding in the pediatric intensive care unit (ICU) setting. Multiple factors contribute to feeding intolerance in the ICU including sepsis, respiratory compromise, and cardiovascular insufficiency. Gastric lactobezoars (GLBs) are thought to be rare, but should be considered when evaluating feeding intolerance. GLBs are a type of bezoar comprised of milk constituents and mucus secretions. They are typically found in the stomach but can be found in other areas of the gastrointestinal tract [1, 2]. GLBs can be asymptomatic, but more commonly present with nonspecific symptoms such as vomiting, abdominal distention, diarrhea, poor weight gain, and gastric outlet obstruction. The most worrisome complication is gastric perforation [3–5]. Conservative management is often successful in treating these bezoars. However, in high-risk patients, more aggressive treatment should be considered. This case presents successful dissolution of a GLB in a medically complex patient using N-acetylcysteine (NAC).

## 2. Case Report

**Figure 1:**
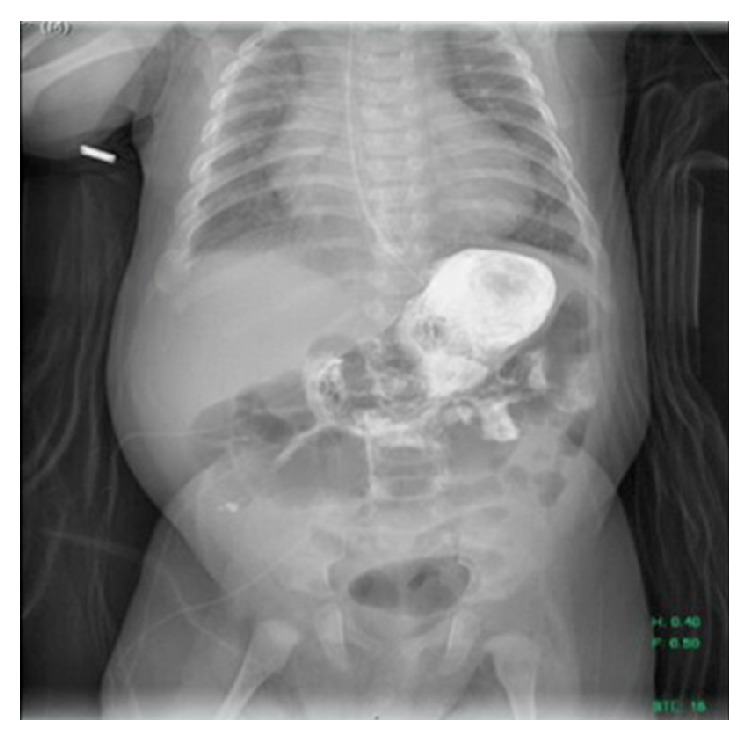
A large ovoid filling defect is noted within the gastric body on this image from an upper gastrointestinal fluoroscopic series.

The patient was a 4-month-old full term male hospitalized since birth with a medical history of trisomy 21, congenital hypothyroidism, failure to thrive, pulmonary hypertension, and complete atrioventricular canal defect with repair complicated by chylothorax requiring chest tube drainage who presented with feeding intolerance. He developed feeding issues at day 18 of life requiring initiation of nasogastric tube feeds. A clinical feeding evaluation by Speech Pathology demonstrated severe behavioral feeding challenges and significantly disorganized feeding skills. Due to his history of chylothorax, he was placed on Enfaport, a casein-based formula high in medium chain triglycerides. The formula was also concentrated to 30 kcal/oz secondary to poor weight gain. His underlying cardiac condition required strict fluid management with multiple diuretics. He remained well from a gastrointestinal perspective except for persistent feeding issues. He did not have emesis, diarrhea, constipation, melena, irritability, or increased gastric residuals. He was tolerating his nasogastric tube feeds well. His physical exam was significant for a distended abdomen. His feeding issues continued and he underwent a videofluoroscopic swallow study (VFSS) at 17 weeks of age. The VFSS showed no evidence of aspiration, but there was concern for gastroesophageal reflux disease. An upper gastrointestinal fluoroscopic series demonstrated a large freely mobile intraluminal ovoid filling defect in the gastric body consistent with a GLB (Figure 1). Delayed gastric emptying into the duodenum was also noted on this exam.

**Figure 2:**
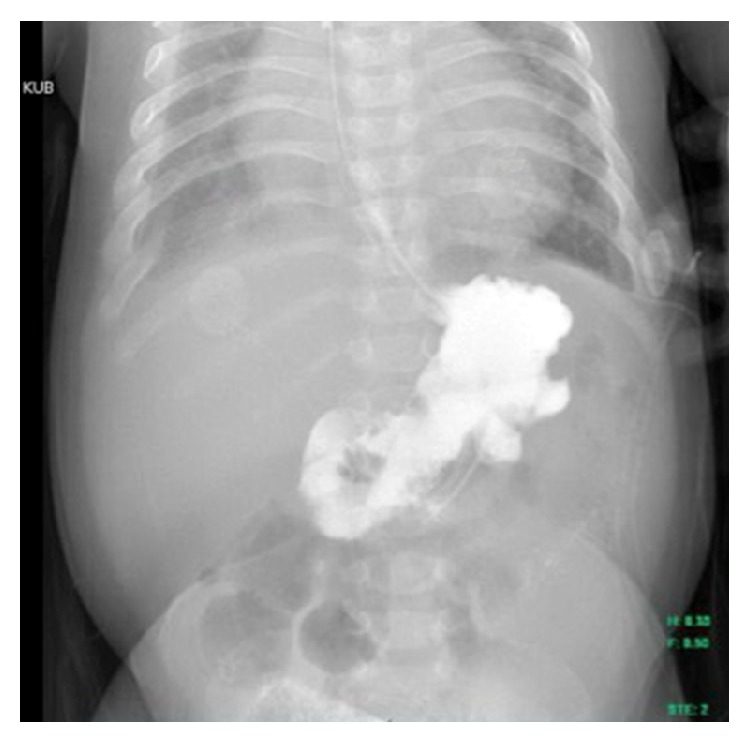
Resolution of the ovoid filling defect on a subsequent upper gastrointestinal fluoroscopic series after treatment with N-acetylcysteine.

Due to concern for adverse outcomes related to the GLB, the decision was made to forgo conservative management and start NAC. Over the course of three days, the patient received 10% NAC at 10 mg/kg/dose diluted in 50 mL of normal saline via nasogastric tube every six hours. The medication was administrated over 30 minutes and the nasogastric tube was clamped for two hours. Gastric contents were aspirated three hours after administration of NAC. Our patient required eight doses of NAC to achieve a clear gastric aspirate. Repeat upper gastrointestinal fluoroscopic imaging demonstrated a complete resolution of the GLB (Figure 2). The infant was subsequently restarted on Enfaport 24 kcal/oz with no further issue or recurrence of the GLB.

## 3. Discussion

A gastric lactobezoar (GLB) is a pathologic mass comprised of inspissated mucous and milk constituents. First described in 1959 by Wolf and Bruce, GLBs are a rare condition with less than 100 pediatric cases reported in the literature since 1975 [6, 7]. They differ from other bezoars such as trichobezoars or medication bezoars by their physical make-up and age of presentation. The majority of GLBs have been reported in infants, the oldest pediatric case being an 8-year-old with cerebral palsy [8]. GLBs are the most common type of bezoar found in infants [9]. Contributing factors toward the pathogenesis of GLBs include dehydration and formulas high in medium chain triglycerides, casein, and caloric density [9]. These risk factors were all found in our patient as he was on concentrated Enfaport, fluid restriction, and multiple diuretics.

GLBs can present in a variety of ways. Less than 5% are asymptomatic. They typically present with a gastrointestinal manifestation including abdominal distension, emesis, gastric residuals, palpable abdominal mass, dehydration, diarrhea, and poor weight gain [6, 10]. Other less common symptoms include respiratory distress, irritability, and lethargy [6].

Though infrequent, serious complications may arise from lactobezoars such as gastric perforation. Two cases of gastric perforation were reported in infants initially suspected of necrotizing enterocolitis. A GLB, as the cause of their symptoms, was discovered after emergent surgery in each patient [3]. Additional reports of gastric perforation have been described [4, 5].

Conservative management includes withholding enteral feeds and starting intravenous fluids. Gastric lavage can also be utilized. This approach results in the resolution of the vast majority of GLBs, though it may take up to a few weeks. [6, 10, 11]. This approach may not be prudent in medically complicated patients prone to complications. Both surgical and endoscopic removals have been reported [12]. Nasogastric administration of N-acetylcysteine (NAC) is a novel, safe, and noninvasive treatment for GLBs. NAC is a mucolytic agent that works by cleaving disulfide bonds in mucoproteins, thereby decreasing mucous viscosity [13]. It is used to treat viscous fecal material in distal ileal obstructive syndrome and, in a nebulized form, to treat mucus secretions in various bronchopulmonary diseases. Its use for the treatment of other types of gastric bezoars has been reported as far back as 1970 [14]. Two recent case reports describe four patients with symptomatic lactobezoars that all resolved after serial nasogastric administrations of NAC [9, 11]. NAC should be considered not only in patients that fail conservative management, but also, as a primary treatment option, for medically complex patients with GLBs.

## 4. Conclusion

Gastric lactobezoars (GLBs) are a rare entity that present with nonspecific symptoms including feeding intolerance. A high index of suspicion should be maintained in patients with known risk factors. Conservative management can result in successful dissolution of the majority of GLBs. However, the required duration of treatment and risk of serious complications may preclude the use of conservative management in specific cases. This case demonstrates successful dissolution of a gastric lactobezoar using N-acetylcysteine. In this single instance, its use was safe and effective. Further studies may be beneficial to outline the safety profile of repeated gastric lavage with this compound, as well as appropriate treatment courses and proof of dissolution.

## Abbreviations

GLB: Gastric lactobezoar
ICU: Intensive care unit
NAC: N-Acetylcysteine
VFSS: Video fluoroscopic swallow study.
